# *N*-(4-Bromo­phen­yl)-2-(2-thien­yl)acetamide

**DOI:** 10.1107/S1600536809053355

**Published:** 2009-12-16

**Authors:** Thais C. M. Nogueira, Marcus V. N. de Souza, James L. Wardell, Solange M. S. V. Wardell, Edward R. T. Tiekink

**Affiliations:** a FioCruz – Fundação Oswaldo Cruz, Instituto de Tecnologia em Farmacos–FarManguinhos, Rua Sizenando Nabuco, 100, Manguinhos, 21041-250 Rio de Janeiro, RJ, Brazil; b Centro de Desenvolvimento Tecnológico em Saúde (CDTS), Fundação Oswaldo Cruz (FIOCRUZ), Casa Amarela, Campus de Manguinhos, Av. Brasil 4365, 21040-900 Rio de Janeiro, RJ, Brazil; c CHEMSOL, 1 Harcourt Road, Aberdeen AB15 5NY, Scotland; d Department of Chemistry, University of Malaya, 50603 Kuala Lumpur, Malaysia

## Abstract

The thienyl ring in the title compound, C_12_H_10_BrNOS, is disordered over two diagonally opposite positions, the major component having a site-occupancy factor of 0.660 (5). The mol­ecule is twisted as evidenced by the dihedral angles of 70.0 (4) and 70.5 (6)° formed between the benzene ring and the two orientations of the disordered thio­phene ring. Linear supra­molecular chains along the *a* axis are found in the crystal structure through the agency of N—H⋯O hydrogen bonding.

## Related literature

For background to the various applications of 2-substituted thio­phenes, see: Campaigne (1984 ▶); Kleemann *et al.* (2006 ▶). For recent biological studies on 2-substituted thio­phenes, see: Lourenço *et al.* (2007 ▶). For the structure of the *N*-(2,6-dimethyl­phen­yl) derivative, see: Ferreira *et al.* (2009 ▶).
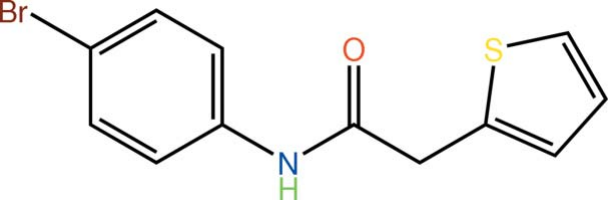


## Experimental

### 

#### Crystal data

C_12_H_10_BrNOS
*M* *_r_* = 296.18
Triclinic, 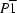

*a* = 4.7517 (2) Å
*b* = 10.7283 (3) Å
*c* = 11.7964 (3) Å
α = 76.419 (2)°
β = 88.437 (2)°
γ = 84.479 (2)°
*V* = 581.82 (3) Å^3^
*Z* = 2
Mo *K*α radiation
μ = 3.69 mm^−1^
*T* = 120 K
0.09 × 0.06 × 0.02 mm

#### Data collection

Nonius KappaCCD area-detector diffractometer
Absorption correction: multi-scan (*SADABS*; Sheldrick, 2003 ▶) *T*_min_ = 0.845, *T*_max_ = 1.000
9757 measured reflections
2045 independent reflections
1847 reflections with *I* > 2σ(*I*)
*R*_int_ = 0.042

#### Refinement

*R*[*F* ^2^ > 2σ(*F* ^2^)] = 0.031
*wR*(*F* ^2^) = 0.115
*S* = 0.98
2045 reflections
161 parameters
1 restraint
H atoms treated by a mixture of independent and constrained refinement
Δρ_max_ = 0.40 e Å^−3^
Δρ_min_ = −0.29 e Å^−3^

### 

Data collection: *COLLECT* (Hooft, 1998 ▶); cell refinement: *DENZO* (Otwinowski & Minor, 1997 ▶) and *COLLECT*; data reduction: *DENZO* and *COLLECT*; program(s) used to solve structure: *SHELXS97* (Sheldrick, 2008 ▶); program(s) used to refine structure: *SHELXL97* (Sheldrick, 2008 ▶); molecular graphics: *ORTEP-3* (Farrugia, 1997 ▶) and *DIAMOND* (Brandenburg, 2006 ▶); software used to prepare material for publication: *publCIF* (Westrip, 2009 ▶).

## Supplementary Material

Crystal structure: contains datablocks global, I. DOI: 10.1107/S1600536809053355/hg2617sup1.cif

Structure factors: contains datablocks I. DOI: 10.1107/S1600536809053355/hg2617Isup2.hkl

Additional supplementary materials:  crystallographic information; 3D view; checkCIF report

## Figures and Tables

**Table 1:** Hydrogen-bond geometry (Å, °)

*D*—H⋯*A*	*D*—H	H⋯*A*	*D*⋯*A*	*D*—H⋯*A*
N1—H1n⋯O1^i^	0.88 (2)	2.00 (2)	2.848 (3)	162 (3)

Symmetry code: (i) 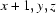.

**Fig. 1.**
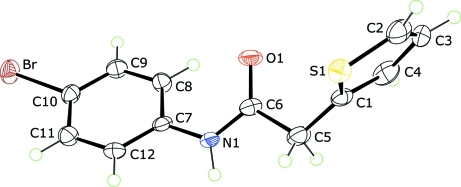
Molecular structure (I) showing atom-labelling scheme and displacement ellipsoids at the 50% probability level. Only the major component of the disordered thienyl ring is shown for reasons of clarity.

**Fig. 2.**
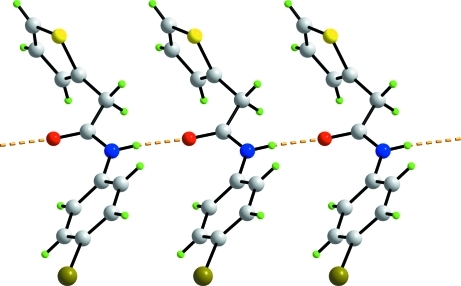
Supramolecular chain in (I) aligned along the a axis and mediated by N–H···O hydrogen bonds (blue dashed lines). Colour code: Br, olive; S, yellow; O, red; N, blue; C, grey; and H, green.

## supplementary crystallographic information

### Comment

The various uses, for example, as dyestuffs, flavour agents, drugs, and inhibitors, have been well documented for 2-substituted thiophenes related to the title compound (Campaigne, 1984). Thiophenes are present in many natural and synthetic products that have a wide range of pharmacological activities (Kleemann *et al.*, 2006). The *in vitro* antimycobacterial activities of a series of *N*-(aryl)-2-thiophen-2-ylacetamide derivatives were recently investigated and encouraging activities were detected for some of these (Lourenço *et al.*, 2007). The search for new drugs having antibacterial activity against Mycobacterium tuberculosis is a vital task due to the increase of multi-drug resistant tuberculosis (MDR-TB) and AIDS cases worldwide, and the increasing resistance to the currently used main line drugs such as isoniazid and rifampin (http://www.who.int/tdr/diseases/tb/default.htm). Recently, we reported the structure of *N*-(2,6-dimethylphenyl)-2-(thiophen-2-yl)acetamide (Ferreira *et al.*, 2009) and as a continuation of these studies, the title thiophene derivative, (I), is described.

The molecular structure of (I), Fig. 1, is twisted as seen in the values of the C6–N1–C7–C8 and S1–C1–C5–C6 torsion angles of 35.0 (5) and 88.4 (4) °, respectively; the S1'–C1–C5–C6 torsion angle for the minor component of the disordered thiophene ring is -89.9 (5) °. The dihedral angle formed between the thiophene and benzene rings is 70.0 (4) °; the equivalent angle involving the minor component of the thiophene ring is 70.5 (6) °. The anti-conformation of the amide group allows for the formation of linear supramolecular chains along the *a* axis *via* N–H···O hydrogen bonding, Fig. 2 and Table 1.

### Experimental

A solution of 4-bromoaniline (2 mmol) and 2-thienylacetyl chloride (2 mmol) in tetrahydrofuran (20 ml) was stirred for 2 h at room temperature, water (30 ml) added and the mixture was extracted with ethyl acetate (2 *x* 20 ml). The combined organic layers were washed with saturated aqueous NaHCO_3_ and brine, dried over MgSO_4_, filtered, and rotary evaporated to give the crude product, (yield 96%), which was recrystallized from EtOH; m.pt.: 411–412 K. CG/MS: m/*z* [*M*]^+.^: 297. ^1^H NMR [500.00 MHz, DMSO-d_6_] δ: 10.30 (1*H*, s, NH), 7.6 (d, 2H, J = 9.0 Hz), 7.48 (d, 2H, J = 9.0 Hz), 7.38, (dd, 1H, J = 4.5 and 2.0 Hz), 6.98–6.96 (m, 2H), 3.87 (s, 2H, CH_2_CO) p.p.m.. ^13^C NMR (125.0 MHz, DMSO-d_6_) δ: 168.1, 138.3, 136.8, 131.5, 126.6, 126.3, 125.0, 121.3, 114.8, 37.4 p.p.m.. IR (KBr, cm^-1^) ν: 1660 (CO).

### Refinement

The C-bound H atoms were geometrically placed (C–H = 0.95–0.99 Å) and refined as riding with *U*_iso_(H) = 1.2*U*_eq_(C). The N–H atom was located in a difference map and refined with the distance restraint N–H = 0.88±0.01 and with *U*_iso_(H) = 1.2*U*_eq_(N). The thienyl ring was disordered with two diagonally opposed positions resolved for the S1 and C4 atoms (the anisotropic displacement parameters for the two components of the C4 atom were constrained to be equal). The major component had a site occupancy factor = 0.660 (5).

### Crystal data

**Table:**

C_12_H_10_BrNOS	*Z* = 2
*M**_r_* = 296.18	*F*(000) = 296
Triclinic, *P*1	*D*_x_ = 1.691 Mg m^−^^3^
Hall symbol: -P 1	Mo *K*α radiation, λ = 0.71073 Å
*a* = 4.7517 (2) Å	Cell parameters from 2592 reflections
*b* = 10.7283 (3) Å	θ = 2.9–27.5°
*c* = 11.7964 (3) Å	µ = 3.69 mm^−^^1^
α = 76.419 (2)°	*T* = 120 K
β = 88.437 (2)°	Block, pale-brown
γ = 84.479 (2)°	0.09 × 0.06 × 0.02 mm
*V* = 581.82 (3) Å^3^	

### Data collection

**Table:**

Enraf–Nonius KappaCCD area-detector diffractometer	2045 independent reflections
Radiation source: Enraf Nonius FR591 rotating anode	1847 reflections with *I* > 2σ(*I*)
10 cm confocal mirrors	*R*_int_ = 0.042
Detector resolution: 9.091 pixels mm^-1^	θ_max_ = 25.0°, θ_min_ = 2.9°
φ and ω scans	*h* = −5→5
Absorption correction: multi-scan (*SADABS*; Sheldrick, 2003)	*k* = −12→12
*T*_min_ = 0.845, *T*_max_ = 1.000	*l* = −14→14
9757 measured reflections	

### Refinement

**Table:**

Refinement on *F*^2^	Primary atom site location: structure-invariant direct methods
Least-squares matrix: full	Secondary atom site location: difference Fourier map
*R*[*F*^2^ > 2σ(*F*^2^)] = 0.031	Hydrogen site location: inferred from neighbouring sites
*wR*(*F*^2^) = 0.115	H atoms treated by a mixture of independent and constrained refinement
*S* = 0.98	*w* = 1/[σ^2^(*F*_o_^2^) + (0.1*P*)^2^] where *P* = (*F*_o_^2^ + 2*F*_c_^2^)/3
2045 reflections	(Δ/σ)_max_ = 0.001
161 parameters	Δρ_max_ = 0.40 e Å^−^^3^
1 restraint	Δρ_min_ = −0.29 e Å^−^^3^

### Special details

**Table:**

Geometry. All s.u.'s (except the s.u. in the dihedral angle between two l.s. planes) are estimated using the full covariance matrix. The cell s.u.'s are taken into account individually in the estimation of s.u.'s in distances, angles and torsion angles; correlations between s.u.'s in cell parameters are only used when they are defined by crystal symmetry. An approximate (isotropic) treatment of cell s.u.'s is used for estimating s.u.'s involving l.s. planes.
Refinement. Refinement of *F*^2^ against ALL reflections. The weighted *R*-factor *wR* and goodness of fit *S* are based on *F*^2^, conventional *R*-factors *R* are based on *F*, with *F* set to zero for negative *F*^2^. The threshold expression of *F*^2^ > 2σ(*F*^2^) is used only for calculating *R*-factors(gt) *etc*. and is not relevant to the choice of reflections for refinement. *R*-factors based on *F*^2^ are statistically about twice as large as those based on *F*, and *R*- factors based on ALL data will be even larger.

### Fractional atomic coordinates and isotropic or equivalent isotropic displacement parameters (Å^2^)

**Table:**

	*x*	*y*	*z*	*U*_iso_*/*U*_eq_	Occ. (<1)
Br	0.27885 (7)	−0.23040 (3)	0.97460 (3)	0.03386 (19)	
O1	0.0821 (5)	0.2338 (2)	0.4702 (2)	0.0338 (6)	
N1	0.5204 (5)	0.1924 (3)	0.5506 (2)	0.0237 (6)	
H1N	0.696 (3)	0.211 (3)	0.540 (3)	0.028*	
S1	0.0537 (8)	0.5801 (3)	0.3373 (3)	0.0278 (6)	0.660 (5)
C1	0.2232 (6)	0.4487 (3)	0.3020 (3)	0.0249 (7)	0.660 (5)
C2	−0.1461 (8)	0.6162 (4)	0.2202 (3)	0.0361 (8)	0.660 (5)
H2	−0.2806	0.6894	0.2040	0.043*	0.660 (5)
C3	−0.1056 (7)	0.5346 (4)	0.1488 (3)	0.0318 (8)	0.660 (5)
H3	−0.2006	0.5420	0.0774	0.038*	0.660 (5)
C4	0.108 (4)	0.4342 (19)	0.2003 (15)	0.035 (4)	0.660 (5)
H4	0.1644	0.3633	0.1668	0.042*	0.660 (5)
S1'	0.1360 (17)	0.4123 (9)	0.1850 (6)	0.0245 (14)	0.340 (5)
C1'	0.2232 (6)	0.4487 (3)	0.3020 (3)	0.0249 (7)	0.340 (5)
C2'	−0.1461 (8)	0.6162 (4)	0.2202 (3)	0.0361 (8)	0.340 (5)
H2'	−0.2772	0.6904	0.2108	0.043*	0.340 (5)
C3'	−0.1056 (7)	0.5346 (4)	0.1488 (3)	0.0318 (8)	0.340 (5)
H3'	−0.2134	0.5474	0.0797	0.038*	0.340 (5)
C4'	0.072 (7)	0.561 (3)	0.323 (3)	0.035 (4)	0.340 (5)
H4'	0.0977	0.5980	0.3879	0.042*	0.340 (5)
C5	0.4454 (7)	0.3645 (4)	0.3784 (3)	0.0351 (8)	
H5A	0.5485	0.4183	0.4176	0.042*	
H5B	0.5828	0.3256	0.3290	0.042*	
C6	0.3296 (6)	0.2578 (3)	0.4702 (3)	0.0251 (7)	
C7	0.4675 (6)	0.0909 (3)	0.6462 (3)	0.0227 (7)	
C8	0.2767 (7)	0.0013 (3)	0.6398 (3)	0.0251 (7)	
H8	0.1806	0.0071	0.5689	0.030*	
C9	0.2278 (7)	−0.0953 (3)	0.7360 (3)	0.0266 (7)	
H9	0.0995	−0.1566	0.7315	0.032*	
C10	0.3667 (7)	−0.1026 (3)	0.8397 (3)	0.0252 (7)	
C11	0.5612 (7)	−0.0172 (3)	0.8476 (3)	0.0279 (7)	
H11	0.6607	−0.0254	0.9181	0.033*	
C12	0.6090 (7)	0.0808 (3)	0.7505 (3)	0.0255 (7)	
H12	0.7389	0.1414	0.7553	0.031*	

### Atomic displacement parameters (Å^2^)

**Table:**

	*U*^11^	*U*^22^	*U*^33^	*U*^12^	*U*^13^	*U*^23^
Br	0.0441 (3)	0.0281 (3)	0.0269 (3)	−0.00963 (17)	0.00026 (16)	0.00104 (16)
O1	0.0170 (11)	0.0346 (14)	0.0425 (15)	−0.0075 (10)	−0.0019 (10)	0.0076 (11)
N1	0.0165 (12)	0.0273 (15)	0.0256 (13)	−0.0067 (11)	0.0018 (11)	−0.0008 (11)
S1	0.0324 (10)	0.0221 (11)	0.0298 (12)	−0.0051 (8)	0.0006 (8)	−0.0068 (9)
C1	0.0191 (15)	0.0207 (17)	0.0320 (18)	−0.0039 (12)	0.0043 (13)	−0.0002 (13)
C2	0.0328 (18)	0.0267 (19)	0.042 (2)	−0.0012 (15)	0.0071 (15)	0.0040 (15)
C3	0.0289 (17)	0.039 (2)	0.0236 (17)	−0.0084 (15)	−0.0003 (13)	0.0034 (15)
C4	0.037 (5)	0.038 (7)	0.036 (6)	−0.007 (4)	0.015 (3)	−0.022 (4)
S1'	0.027 (2)	0.029 (3)	0.019 (2)	−0.0057 (17)	0.0037 (17)	−0.007 (2)
C1'	0.0191 (15)	0.0207 (17)	0.0320 (18)	−0.0039 (12)	0.0043 (13)	−0.0002 (13)
C2'	0.0328 (18)	0.0267 (19)	0.042 (2)	−0.0012 (15)	0.0071 (15)	0.0040 (15)
C3'	0.0289 (17)	0.039 (2)	0.0236 (17)	−0.0084 (15)	−0.0003 (13)	0.0034 (15)
C4'	0.037 (5)	0.038 (7)	0.036 (6)	−0.007 (4)	0.015 (3)	−0.022 (4)
C5	0.0228 (16)	0.034 (2)	0.041 (2)	−0.0067 (14)	0.0030 (14)	0.0078 (16)
C6	0.0191 (15)	0.0229 (18)	0.0329 (19)	−0.0039 (13)	0.0040 (13)	−0.0053 (15)
C7	0.0141 (13)	0.0267 (17)	0.0264 (16)	−0.0020 (12)	0.0051 (12)	−0.0050 (13)
C8	0.0278 (16)	0.0224 (17)	0.0257 (16)	−0.0059 (13)	−0.0032 (13)	−0.0048 (13)
C9	0.0254 (16)	0.0235 (17)	0.0317 (17)	−0.0058 (13)	0.0017 (13)	−0.0070 (14)
C10	0.0265 (16)	0.0225 (17)	0.0245 (16)	−0.0022 (13)	0.0025 (13)	−0.0017 (13)
C11	0.0263 (16)	0.0309 (19)	0.0274 (17)	−0.0025 (14)	−0.0008 (13)	−0.0086 (14)
C12	0.0185 (14)	0.0278 (18)	0.0318 (17)	−0.0061 (12)	0.0001 (12)	−0.0087 (14)

### Geometric parameters (Å, °)

**Table:**

Br—C10	1.907 (3)	C2'—C3'	1.348 (5)
O1—C6	1.228 (4)	C2'—C4'	1.59 (3)
N1—C6	1.352 (4)	C2'—H2'	0.9500
N1—C7	1.407 (4)	C3'—H3'	0.9500
N1—H1N	0.875 (10)	C4'—H4'	0.9500
S1—C2	1.647 (5)	C5—C6	1.514 (5)
S1—C1	1.687 (4)	C5—H5A	0.9900
C1—C4	1.381 (17)	C5—H5B	0.9900
C1—C5	1.493 (5)	C7—C12	1.396 (4)
C2—C3	1.348 (5)	C7—C8	1.399 (4)
C2—H2	0.9500	C8—C9	1.377 (5)
C3—C4	1.439 (19)	C8—H8	0.9500
C3—H3	0.9500	C9—C10	1.388 (5)
C4—H4	0.9500	C9—H9	0.9500
S1'—C1'	1.594 (8)	C10—C11	1.382 (5)
S1'—C3'	1.641 (9)	C11—C12	1.391 (5)
C1'—C4'	1.42 (3)	C11—H11	0.9500
C1'—C5	1.493 (5)	C12—H12	0.9500
			
C6—N1—C7	126.1 (3)	C1—C5—C6	113.6 (3)
C6—N1—H1n	117 (2)	C1'—C5—H5A	108.8
C7—N1—H1n	117 (2)	C1—C5—H5A	108.8
C2—S1—C1	93.6 (2)	C6—C5—H5A	108.8
C4—C1—C5	129.6 (8)	C1'—C5—H5B	108.8
C4—C1—S1	108.3 (8)	C1—C5—H5B	108.8
C5—C1—S1	122.0 (3)	C6—C5—H5B	108.8
C3—C2—S1	115.4 (3)	H5A—C5—H5B	107.7
C3—C2—H2	122.3	O1—C6—N1	123.6 (3)
S1—C2—H2	122.3	O1—C6—C5	122.0 (3)
C2—C3—C4	107.7 (7)	N1—C6—C5	114.5 (3)
C2—C3—H3	126.2	C12—C7—C8	119.5 (3)
C4—C3—H3	126.2	C12—C7—N1	118.5 (3)
C1—C4—C3	114.9 (11)	C8—C7—N1	122.1 (3)
C1—C4—H4	122.6	C9—C8—C7	120.1 (3)
C3—C4—H4	122.6	C9—C8—H8	119.9
C1'—S1'—C3'	94.5 (5)	C7—C8—H8	119.9
C4'—C1'—C5	126.1 (14)	C10—C9—C8	119.7 (3)
C4'—C1'—S1'	114.4 (14)	C10—C9—H9	120.2
C5—C1'—S1'	119.4 (4)	C8—C9—H9	120.2
C3'—C2'—C4'	105.2 (11)	C11—C10—C9	121.3 (3)
C3'—C2'—H2'	127.4	C11—C10—Br	119.6 (2)
C4'—C2'—H2'	127.4	C9—C10—Br	119.1 (2)
C2'—C3'—S1'	117.9 (4)	C10—C11—C12	118.9 (3)
C2'—C3'—H3'	121.1	C10—C11—H11	120.5
S1'—C3'—H3'	121.1	C12—C11—H11	120.5
C1'—C4'—C2'	108.0 (19)	C7—C12—C11	120.4 (3)
C1'—C4'—H4'	126.0	C7—C12—H12	119.8
C2'—C4'—H4'	126.0	C11—C12—H12	119.8
C1'—C5—C6	113.6 (3)		
			
C2—S1—C1—C4	−2.5 (10)	C7—N1—C6—O1	−1.5 (5)
C2—S1—C1—C5	−179.0 (3)	C7—N1—C6—C5	178.7 (3)
C1—S1—C2—C3	0.8 (4)	C1'—C5—C6—O1	9.4 (5)
S1—C2—C3—C4	1.0 (9)	C1—C5—C6—O1	9.4 (5)
C5—C1—C4—C3	179.8 (7)	C1'—C5—C6—N1	−170.8 (3)
S1—C1—C4—C3	3.6 (17)	C1—C5—C6—N1	−170.8 (3)
C2—C3—C4—C1	−3.0 (16)	C6—N1—C7—C12	−144.4 (3)
C3'—S1'—C1'—C4'	−0.6 (19)	C6—N1—C7—C8	35.0 (5)
C3'—S1'—C1'—C5	178.7 (3)	C12—C7—C8—C9	0.5 (5)
C4'—C2'—C3'—S1'	0.1 (13)	N1—C7—C8—C9	−178.9 (3)
C1'—S1'—C3'—C2'	0.3 (6)	C7—C8—C9—C10	0.6 (5)
C5—C1'—C4'—C2'	−178.6 (8)	C8—C9—C10—C11	−2.2 (5)
S1'—C1'—C4'—C2'	1(3)	C8—C9—C10—Br	176.1 (2)
C3'—C2'—C4'—C1'	0(2)	C9—C10—C11—C12	2.6 (5)
C4'—C1'—C5—C6	89.3 (18)	Br—C10—C11—C12	−175.7 (2)
S1'—C1'—C5—C6	−89.9 (5)	C8—C7—C12—C11	0.0 (5)
C4—C1—C5—C6	−87.3 (12)	N1—C7—C12—C11	179.4 (3)
S1—C1—C5—C6	88.4 (4)	C10—C11—C12—C7	−1.5 (5)

### Hydrogen-bond geometry (Å, °)

**Table:**

*D*—H···*A*	*D*—H	H···*A*	*D*···*A*	*D*—H···*A*
N1—H1n···O1^i^	0.875 (17)	2.00 (2)	2.848 (3)	162 (3)

Symmetry codes: (i) *x*+1, *y*, *z*.

## References

[bb1] Brandenburg, K. (2006). *DIAMOND* Crystal Impact GbR, Bonn, Germany.

[bb2] Campaigne, E. (1984). In: *Comprehensive Heterocyclic Chemistry*, Vol. 4, edited by A. R. Katritzky & Rees, C. W. pp. 863–934. Oxford: Pergamon.

[bb3] Farrugia, L. J. (1997). *J. Appl. Cryst.* **30**, 565.

[bb4] Ferreira de Lima, M., de Souza, M. V. N., Tiekink, E. R. T., Wardell, J. L. & Wardell, S. M. S. V. (2009). *Acta Cryst.* E**65**, o3203. 10.1107/S1600536809049782 PMC2972164 21578912

[bb5] Hooft, R. W. W. (1998). *COLLECT* Nonius BV, Delft, The Netherlands.

[bb6] Kleemann, A., Engel, J. B., Kutscher, B. & Reichert, D. (2006). In *Pharmaceutical Substances* New York, Stuttgart: Georg Thieme Verlag.

[bb7] Lourenço, M. C. S., Vicente, F. R., Henriques, M., das, G. M. de O., Candéa, A. L. P., Gonçalves, R. S. B., Nogueira, T. C. M., Ferreira, M. de L. & de Souza, M. V. N. (2007). *Bioorg. Med. Chem. Lett.* **17**, 6895–6898.

[bb8] Otwinowski, Z. & Minor, W. (1997). *Methods in Enzymology*, Vol. 276, *Macromolecular Crystallography*, Part A, edited by C. W. Carter Jr & R. M. Sweet, pp. 307–326. New York: Academic Press.

[bb9] Sheldrick, G. M. (2003). *SADABS* Bruker AXS Inc., Madison, Wisconsin, USA.

[bb10] Sheldrick, G. M. (2008). *Acta Cryst.* A**64**, 112–122. 10.1107/S0108767307043930 18156677

[bb11] Westrip, S. P. (2009). *publCIF* In preparation.

